# Prevalence of intimate partner violence among Indian women and their determinants: a cross-sectional study from national family health survey – 5

**DOI:** 10.1186/s12905-024-03204-x

**Published:** 2024-06-22

**Authors:** Sayantani Manna, Damini Singh, Manish Barik, Tanveer Rehman, Shishirendu Ghosal, Srikanta Kanungo, Sanghamitra Pati

**Affiliations:** Division of Health Research, ICMR-Regional Medical Research Centre, Bhubaneswar, Odisha India

**Keywords:** Intimate partner violence, Sexual violence, Physical violence, NFHS-5, India

## Abstract

**Introduction:**

Intimate partner violence (IPV) can be described as a violation of human rights that results from gender inequality. It has arisen as a contemporary issue in societies from both developing and industrialized countries and an impediment to long-term development. This study evaluates the prevalence of IPV and its variants among the empowerment status of women and identify the associated sociodemographic parameters, linked to IPV.

**Methods:**

This study is based on data from the National Family Health Survey (NFHS) of India, 2019-21 a nationwide survey that provides scientific data on health and family welfare. Prevalence of IPV were estimated among variouss social and demographic strata. Pearson chi-square test was used to estimate the strength of association between each possible covariate and IPV. Significantly associated covariates (from univariate logistic regression) were further analyzed through separate bivariate logistic models for each of the components of IPV, viz-a-viz sexual, emotional, physical and severe violence of the partners.

**Results:**

The prevalence of IPV among empowered women was found to be 26.21%. Among those who had experienced IPV, two-thirds (60%) were faced the physical violence. When compared to highly empowered women, less empowered women were 74% more likely to face emotional abuse. Alcohol consumption by a partner was established to be attributing immensely for any kind of violence, including sexual violence [AOR: 3.28 (2.83–3.81)].

**Conclusions:**

Our research found that less empowered women experience all forms of IPV compared to more empowered women. More efforts should to taken by government and other stakeholders to promote women empowerment by improving education, autonomy and decision-making ability.

**Supplementary Information:**

The online version contains supplementary material available at 10.1186/s12905-024-03204-x.

## Introduction

Domestic violence is one of the emerging problems in recent years in both low- and middle-income as well as high-income countries. Gender-based violence, another leading public health problem identified in 1996, is a matter of human rights rooted in gender inequality [1]. The Sustainable Development Goals (SDG) from 2015, also recognized the importance of gender-based violence, which is an advance step to eliminate gender inequality and women empowerment [2, 3]. Intimate partner violence (IPV) is recognized as the most common gender-based violence, which is mostly used as synonymously as domestic or spousal violence but conceptually a subtle difference is present [4]. IPV affect general health and reproductive health of women, causing chronic pain, injuries, fractures, disabilities, unwanted pregnancy and over expose to contraceptive pills, increasing vulnerability to sexually transmitted diseases [5]. Such physical and mental strains gradually bring about in the form of post-traumatic stress disorder (PTSD), anxiety, phobia, depression, alcohol abuse etc [6]. 

IPV has become a global public health problem with the consequences of premature deaths and injuries [7]. World Health Organization (WHO) has recognized IPV as a “global hidden epidemic” [8, 9]. Worldwide, one-third of the women have experienced IPV [3]. Due to stigma and fear Intimate Partner violence (IPV) on married women remain unreported in India [10]. IPV has been recognized as a criminal offence under Indian Penal Code 498-A since 1983. Victims are offered civil protection under the Protection of Women from Domestic Violence Act (PWDVA) 2005, which covers all forms of physical, mental, verbal, sexual and economic violence (unlawful dowry demands), including marital rape and harassment etc [11–13]. According to the National Crime Record Bureau’s report, the rate of total crime per lakh ( per lakh defined in the Indian numbering system as equal to one hundred thousand) in the women population is 56.5 [14].

Evidence suggests IPV is associated with low socioeconomic status and unemployment. Indian-employed women faced IPV at a lower rate [15], while other researchers have identified it as an increased risk of violence [16]. Other studies illustrated little consistency between women empowerment and violence across varying cultures, where educational attainment, income, decision-making, and contextual factors all play vital roles individually [17–19]. On the contrary empowered women and following economic independence act as a shield to domestic violence in high-income countries [20]. Consequently, women’s empowerment would continue to be perceived as a “zero-sum” game with politically robust beneficiaries and weak losers if it was advocated as a goal in and off itself [22]. There may be present specific association and management techniques for each sort of IPV which must thus be researched independently [15]. Hence, in this study, we estimated the prevalence of different IPV categories against empowerment status of women and determined the sociodemographic behaviour associated with IPV.

## Methods

### Overview of data

India is home for more than 1.4 billion population, making this country the second-most populous country in the world [23]. The National Family Health Survey-5 (NFHS-5), which was conducted in all 28 states and 8 union territories of the country, is representative at the national and state/UT levels, adopted in each survey round. A two-stage sampling was done to choose villages and census enumeration blocks from districts in rural and urban regions, respectively. From June 2019 to April 2021, data were collected using CAPI. (Computer-Assisted Personal Interview) with an internal scheduling and adequate maintenance of respondent anonymity. The NFHS-5 methodology has been extensively explained and published elsewhere, including the methods for choosing households and data collection [24].

### Study population and study design

The design for this research is comparable to a cross-sectional study because the secondary data used here is collected during the two phases of NFHS-5: from June 17, 2019, to January 30, 2020, and from January 2, 2020, to April 30, 2021. Women who lived with their spouses or partners and experienced any event of domestic abuse, ever till the day of the interview, were included. The included observations were then the subject of secondary data analysis.

### Sample size

**Fig. 1:**
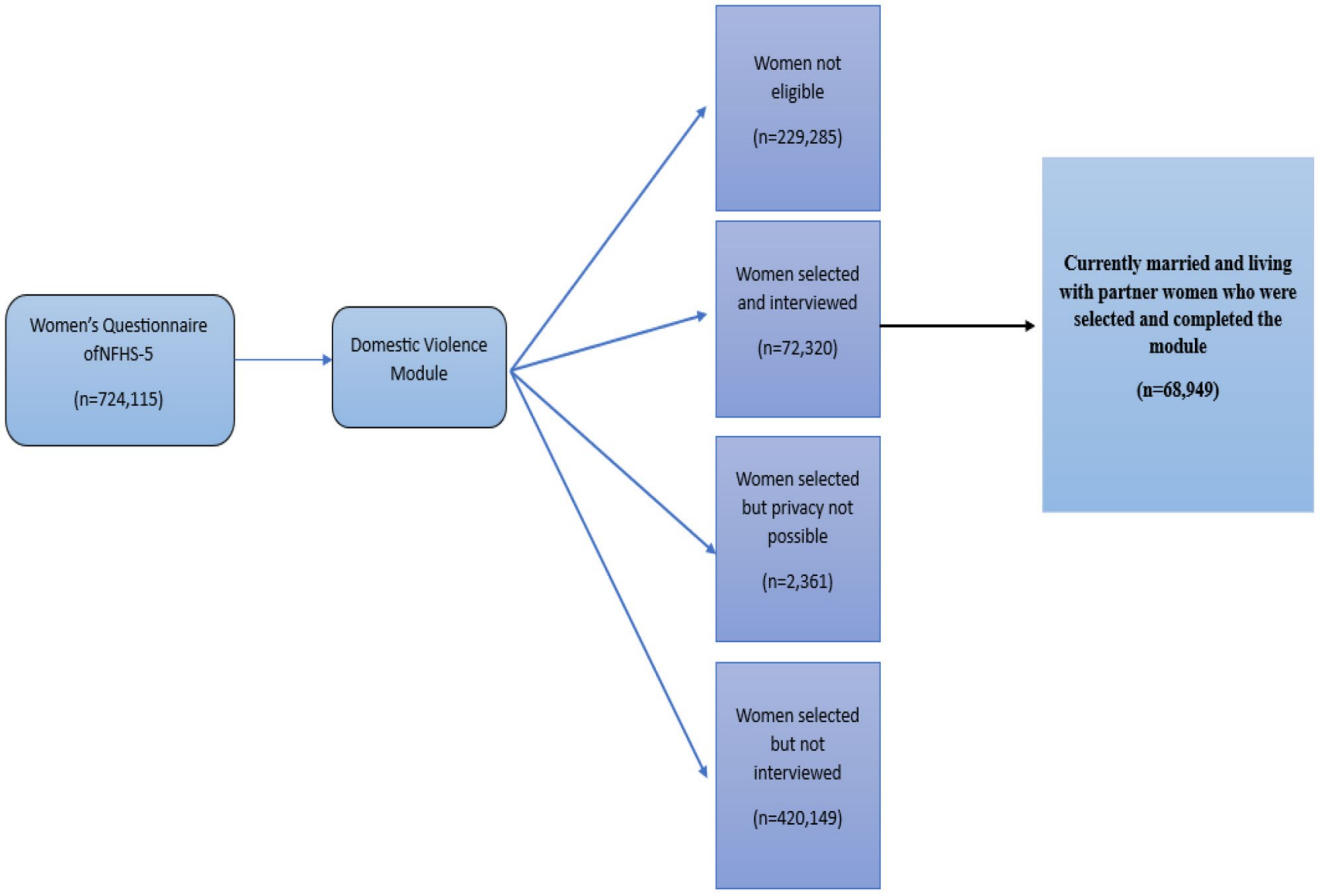
Flow diagram of sample selection from the women’s questionnaire of the NFHS-5

Among the 724,115 women interviewed during the NFHS-5, information was acquired from “never-married” or “ever-married” women aged 18–49 years on their experience of violence committed by their present and previous spouses. Only participants who lived with a partner (married or unmarried) were included in this study **(**Fig. 1**)**. As a result, 68,949 women formed the ultimate sample size.

### Independent variables

The current study focused on the sociodemographic covariates like age, residence (rural/urban), caste, respondent educational qualification, partner’s educational qualification, religion (four categories: Hindu, Muslim, Christian and other religions), wealth index (five quintiles: poorest, poorer, middle, richer and richest quintile), and women empowerment (three categories: low, medium and high ). Another two sets of covariates were the partner’s habit of alcohol consumption and partner controlling behaviour, both dichotomous, grouped as ‘yes’ or ‘no’.

Levels of women’s empowerment were assessed using three indicators: (1) women’s decision-making ability for the household (including access to healthcare, household purchasing and freedom to visit relatives, spending husband earnings, beating wife refuse to have sex), (2) beating indicators(beating the child, wife when argues or refuse to have sex etc.) (3)controlling indicators (includes if allowed to go to market, health facility, outside the village, is justified if went outside without telling), and (4) five economic indicators explaining ownership of the land, house, working status, having a bank account and if owns a mobile phone. All the selected variables are coded into binary variables 0 and 1. Binary variables were included in the composite index to guarantee consistency, while ordinal variables were recoded into binary variables. Table A1 in the supplementary file describes the final variables and their recorded values.

During principal component analysis (PCA), scree plots were examined to determine the number of components to be retained. The scree plot shows that only five components’ eigenvalue is more than 1, which were further processed [4, 19, 25]. The Kaiser-Meyer-Olkin (KMO) measure of sampling adequacy (greater than 0.04 in the PCA) analysis indicates that the sample sizes in this study were appropriate for PCA (Table A2 in the supplementary file). For all components, Bartlett’s test of sphericity confirms that the selected markers of women’s empowerment were intercorrelated. Furthermore, the reliability coefficient (Cronbach’s alpha score:0.60–0.79) demonstrates an adequate component correlation level. We utilized the first component only after loadings and computing component scores, and the index scores were then divided into quintiles (low, medium, and high). Finally, for each selected nation, an overall index of women’s empowerment was built with three ordered categories: low, medium, and high, where ‘low’ indicated women had lower employment and ‘high’ meant women had more empowerment.

### Outcome characteristics: intimate partner violence status

In NFHS-5, a series of questions were asked to collect information on violence committed by the partners, including husbands. It also examines four types of violence faced by women: physical, sexual, emotional, and severe. The level of violence was determined by asking all “ever-married” women if their husbands had ever done the following to them:

#### Physical violence

The IPVs which include any physical violence inflicted on a woman by her husband/partner, which provides for: (a) ever slapped; (b) arm twisted /hair pulled; (c) pushed, shaken/had something thrown at them; (d) punched with a fist or hit by something harmful; (e) kicked/dragged; (f) strangled /brunt; (g) threatened with any weapon.

#### Sexual violence

The Sexual IPVs were captured by three questions in the dataset: (a) physically forced to have sexual intercourse; (b) physically forced to perform any other sexual acts (c) forced you with threats / in any other way to perform sexual acts.

#### Emotional violence

Emotional violence recorded by these questions (a) ever having been said /done something to humiliate you in front of others, b) threatened to hurt /harm you or someone close to you, c) insulted you/make you feel bad about yourself.

#### Severe violence

Severe violence includes physical acts like beatings, choking, burning, and using weapons, as well as sexual violence [5, 26]. NFHS-5 asks specific questions to gather this information are a) ever bruises, b) eye injuries, sprains, dislocations or burns, c) severe burns, d) wounds, broken bones, broken teeth or others.

The answer was classified as “never” if the response was “frequently”, “occasionally”, or “yes but not in the previous 12 months”. Except for ‘never,’ all responses to questions on IPVs indicated prior exposure to physical, sexual, emotional, or serious violence. For simplicity, all responses except ‘never’ were coded as Yes = 1 but never as No = 0.

### Statistical analysis

**Fig. 2:**
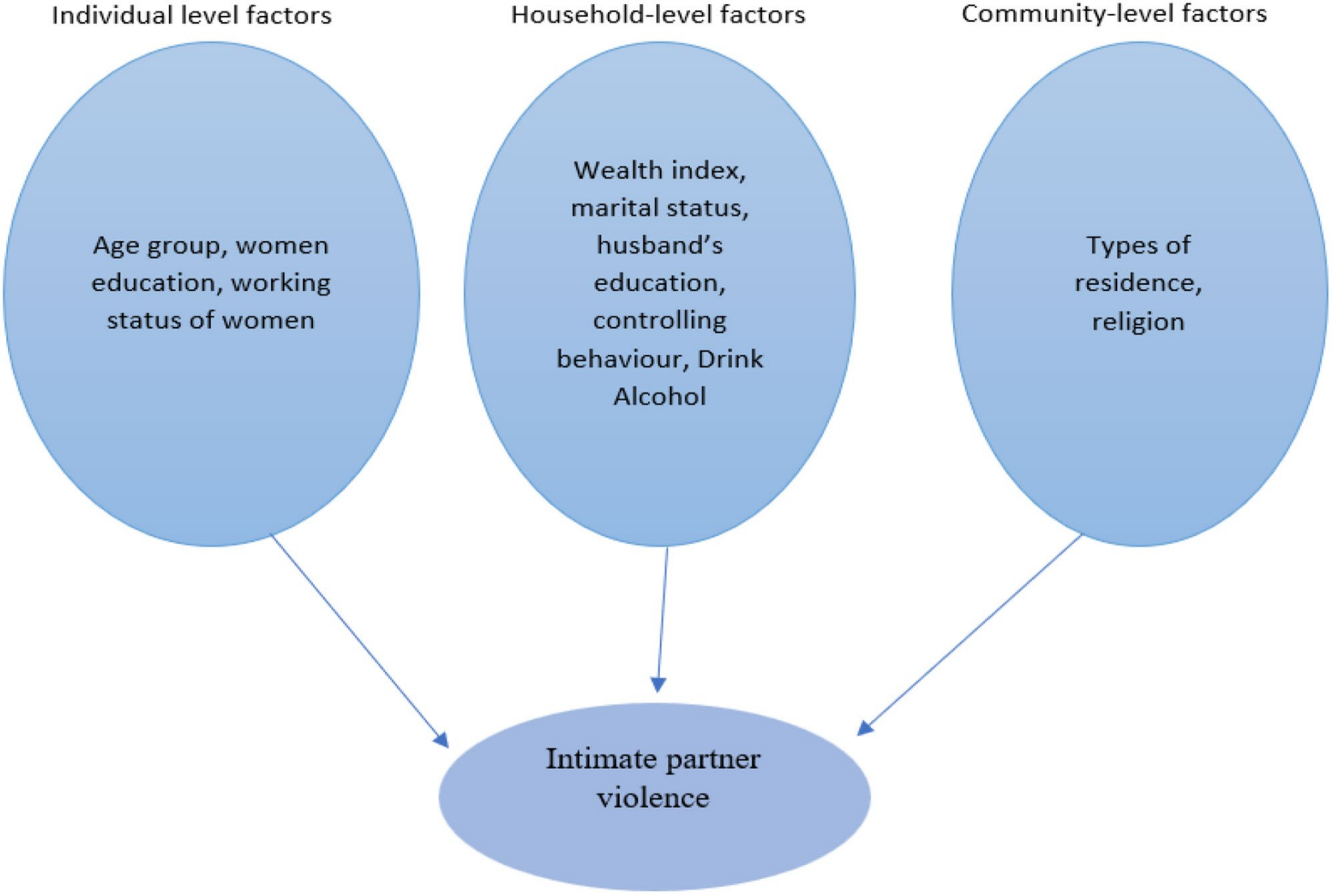
Conceptual framework for the determinants of intimate partner violence

Data analysis was conducted in STATA v17.0 (Stata Corp., Texas). The Fig. 2 below presents a conceptual framework for predicting the socioeconomic determinants of IPV in India. Using this framework, IPV can be characterized as a function of the individual, household, and community variables (Fig. 2**)**. We also analyzed weighted profiles of various IPVs among the sociodemographic and expressed them in numbers and proportions. Distribution of the number of IPV among other categorical was presented as frequencies and association in p-value (< 0.002). To account for the complex survey design, we utilized the domestic violence weighting variable (d005) provided in the NFHS data and applied the survey command (svy), which enabled us to weight the data accurately.

For each independent variable, we performed univariate analysis (Table A3) and incorporated the variables with significant p-values to the multivariable logistic regression model. To assess the appropriateness of the model fit, we utilized two statistical tests: the AIC BIC test and the Hosmer-Lemeshow test. The diminishing values of AIC and BIC suggest that the model is well-suited for the analysis. Moreover, the Hosmer-Lemeshow test yielded a p-value of > 0.05, which reinforces our conclusion that the model is a suitable fit for this analysis. These preliminary models aimed to establish whether any factors should not be regarded as potential covariates for IPV in the multivariate analysis.

## Results

**Fig. 3:**
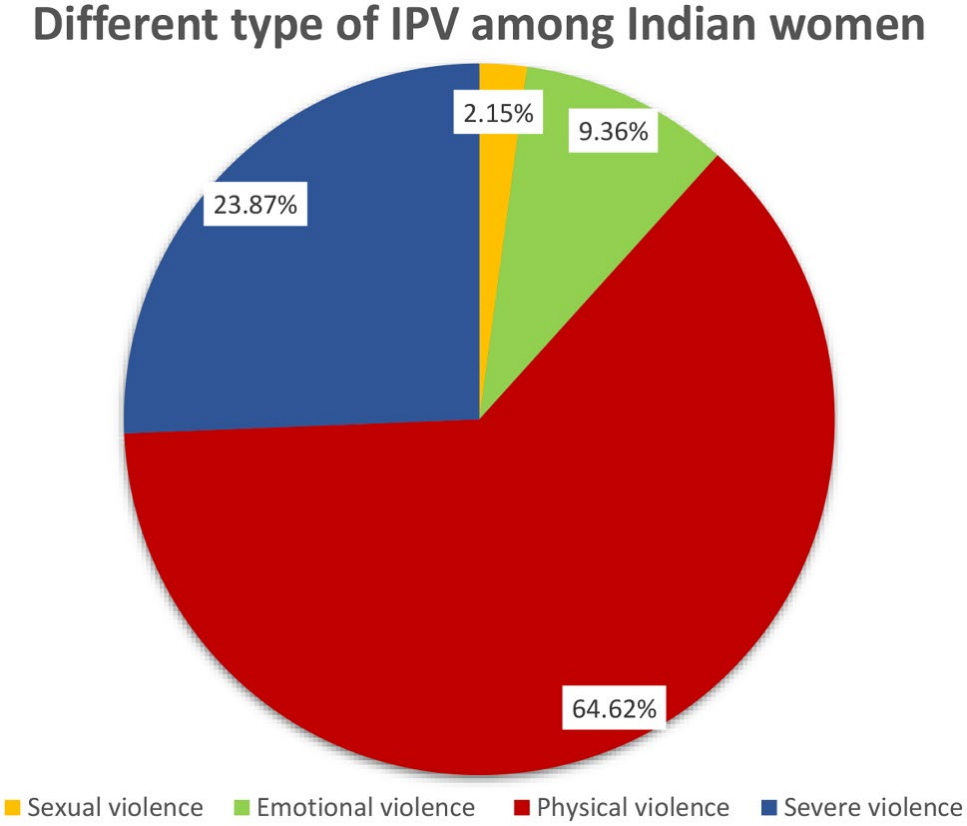
Distribution of various form of IPV among Indian women

Among the 68,949 women in the study, 26.21% (18,074) experienced intimate partner abuse. Most of them belonged to > 35 years of age (40%), and 46% of women completed secondary-level education [Table A3 (Supplementary file)]. Among 26.21% of women who faced any kind of violence, 60% (11,679) experienced physical violence, 23.87% (4,314) were physically injured due to severe IPV, 2.15% experienced sexual violence, and 9.54% experienced emotional violence (Fig. 3).

Table 1 shows the sociodemographic profile, which is further classified by the type of violence experienced. A prevalence of 28.39%, among women aged > 35 years was observed for IPV from their partner. In rural areas have the higher incidence of physical IPV at 26%, compared to urban areas. Women belongs to SC caste had the experienced the highest prevalence of IPV. Women with no formal education (39.03%) and less empowered (37.81%) were the most vulnerable to violence. Similarly, 35% of women who didn’t have any formal education had experienced physical abuse by their partner. When the partner is highly educated, IPV was 19% compared to no formal education (41.60%). IPV was almost equally prevalent among Hinduism (27%) and Muslim women (25%) [physical violence (Hindu: 24.40%; Muslim: 21.31%); emotional violence (Hindu: 11.61%; Muslim: 10.94%)]. In the southern region of India, 30% of women have reported experiencing violence.

**Table 1:** Sociodemographic profile of the participants segregated by type of violence

Background characteristics		Types of violence				
		Emotional n (%) *n* = 7,797 (11.31)	Physical n (%) *n* = 16,121 (23.38)	Sexual n (%) *n* = 3,341 (4.85)	Severe n (%) *n* = 3,892 (5.64)	Total n (%) *n* = 18,254 (26.21)
**Age Group**	**15–24 years** **(** ***N*** **= 14,566)**	1061 (6.14)	2121 (12.26)	480 (2.78)	420 (2.43)	2513 (14.53)
	**25–34 years** **(** ***N*** **= 26,431)**	2786 (12.27)	5781 (25.47)	1199 (5.29)	1306 (5.76)	6486 (28.57)
	**≥ 35 years** **(** ***N*** **= 27,952)**	3950 (13.82)	8219 (28.39)	1661 (5.74)	2166 (7.48)	9256 (31.97)
**Residence**	**Urban** **(** ***N*** **= 17,191)**	1988 (8.99)	4030 (18.22)	751 (3.39)	874 (3.95)	4654 (21.04)
	**Rural** **(** ***N*** **= 51,758)**	5809 (12.41)	12,090 (25.82)	2590 (5.53)	3018 (6.45)	13,601 (29.05)
**Caste**	**Scheduled Caste** **(** ***N*** **= 12,912)**	2010 (13.80)	4111 (28.22)	824 (5.66)	1060 (7.29)	4560 (31.31)
	**Scheduled Tribe** **(** ***N*** **= 13,415)**	744 (11.96)	1585 (25.49)	304 (4.90)	351 (5.64)	1778 (28.58)
	**Other Backward Class** **(** ***N*** **= 26,288)**	3284 (11.41)	7285 (24.70)	1393 (4.72)	1784 (6.05)	8159 (27.67)
	**None of the casts** **(** ***N*** **= 12,592)**	1410 (9.44)	2509 (16.80)	594 (3.98)	532 (3.57)	3011 (20.17)
**Respondent Educational Attainment**	**No formal education** **(** ***N*** **= 17,798)**	2787 (16.60)	5982 (35.64)	1282 (7.64)	1601 (9.53)	6552 (39.03)
	**Completed primary education** **(** ***N*** **= 9,070)**	1142 (13.55)	2500 (20.52)	572 (6.79)	664 (7.87)	2783 (33.02)
	**Completed secondary education** **(** ***N*** **= 32,102)**	3428 (10.45)	6731 (20.52)	1327.25 (4.05)	1480 (4.51)	7819 (23.84)
	**Higher secondary and above** **(** ***N*** **= 9,979)**	440 (4.03)	907 (8.30)	159 (1.46)	148 (1.36)	1101 (10.08)
**Partner’s Educational Attainment**	**No formal education** **(** ***N*** **= 10,353)**	1995 (18.30)	4103 (37.65)	918 (8.43)	1197 (10.99)	4534 (41.60)
	**Completed primary education** **(** ***N*** **= 8,805)**	1458 (15.96)	3029 (33.15)	695 (7.61)	777 (8.50)	3390 (37.10)
	**Completed secondary education** **(** ***N*** **= 32,598)**	3827 (12.18)	7901 (25.16)	1568 (4.99)	1724 (5.49)	9013 (28.70)
	**Higher secondary and above** **(** ***N*** **= 8,585)**	674 (7.59)	1422 (16.01)	229 (2.57)	270 (3.04)	1698 (19.12)
**Religion**	**Hindu** **(** ***N*** **= 51,628)**	6257 (11.61)	13,154 (24.40)	2580 (4.79)	3176 (5.89)	14,805 (27.46)
	**Muslim** **(** ***N*** **= 8,527)**	1247 (10.94)	2429 (21.31)	653 (5.73)	594 (5.21)	2816 (24.71)
	**Christian** **(** ***N*** **= 5,224)**	164 (8.57)	279 (14.57)	51 (2.69)	70 (3.67)	340 (17.78)
	**Other religions** **(** ***N*** **= 3,570)**	129 (7.45)	260 (14.99)	58 (3.32)	52 (3.00)	294 (16.97)
**Wealth Index**	**Poorest quintile** **(** ***N*** **= 14,719)**	1997 (15.80)	4101 (32.46)	1068 (8.46)	1130 (8.94)	4583 (36.27)
	**Poorer quintile** **(** ***N*** **= 15,387)**	1886) (13.31	4145 (29.25)	906 (6.40)	989 (6.98)	4593 (32.41)
	**Middle quintile** **(** ***N*** **= 14,310)**	1735 (11.88)	3498 (23.96)	613 (4.20)	856 (5.86)	3965 (27.16)
	**Richer quintile** **(** ***N*** **= 13,077)**	1344 (9.20)	2733 (18.70)	481 (3.29)	614 (4.20)	3149 (21.56)
	**Richest quintile** **(** ***N*** **= 11,456)**	836 (6.46)	1644 (12.72)	273 (2.11)	303 (2.35)	1964 (15.19)
**Region**	**North** **(** ***N*** **= 13,401)**	373 (6.92)	719 (13.32)	174 (3.23)	183 (3.40)	861 (15.94)
	**Central** **(** ***N*** **= 14,734)**	760 (9.85)	1933 (25.04)	349 (4.52)	435 (5.64)	2129 (27.58)
	**East** **(** ***N*** **= 11,264)**	2646 (13.64)	5384 (27.76)	1351 (6.97)	1327 (6.84)	6049 (31.19)
	**North-east** **(** ***N*** **= 10,600)**	353 (9.19)	885 (23.03)	193 (5.01)	199 (5.17)	988 (25.69)
	**West** **(** ***N*** **= 6,833)**	1272 (8.69)	2394 (16.36)	525 (3.58)	571 (3.90)	2843 (19.42)
	**South** **(** ***N*** **= 1,367)**	2188 (13.66)	4334 (27.06)	642 (4.01)	1039 (6.49)	4869 (30.40)
**Women empowerment**	**Less** **Empowered** **(** ***N*** **= 19,875)**	3875 (17.94)	7162 (33.15)	1780 (8.24)	2047 (9.48)	8168 (37.81)
	**Medium Empowered** **(** ***N*** **= 20,886)**	2274 (11.90)	4983 (26.08)	912 (4.77)	1047 (5.48)	5649 (29.56)
	**High** **Empowered** **(** ***N*** **= 18,862)**	1661 (8.78)	4061 (21.48)	651 (3.44)	799 (4.22)	4543 (24.02)
**Partner drink Alcohol**	**Yes** **(** ***N*** **= 16,360)**	3316 (24.21)	6481 (47.31)	1648 (12.03)	2054 (14.99)	7020 (51.24)
	**No** **(** ***N*** **= 44,120)**	4671 (9.98)	10,032 (2)	1775 (3.79)	1933 (4.13)	11,679 (24.97)
**Partner controlling behaviours**	**Yes** **(** ***N*** **= 24,727)**	6265 (25.14)	10,716 (43.00)	2791 (11.20)	3197 (12.83)	12,249 (49.15)
	**No** **(** ***N*** **= 44,222)**	1533 (4.48)	5405 (12.28)	550 (1.25)	695 (1.58)	6006 (13.64)

The distribution of sampled women based on their background characteristics has been presented in Table A4. The chi-square test is used to assess the strength of association between each socioeconomic variable, and the p-values are provided in the last column of Table A4. Multivariate regression (Table 2) showed a higher chance of experiencing severe IPV among the 25–35 years age-group than the 35–49 years age group with AOR 2.18 (95%CI: 1.69–2.80) in comparison with 15–24 years age group. Respondents who didn’t have any formal education had higher likelihood [AOR = 1.65 (95% CI = 1.35–2.02)] of facing physical violence than women having more than secondary education. Partners with no formal education were significantly associated with any form of violence compared to the highly educated partners. There was 52% greater likelihood among the less empowered women of facing more emotional violence than the highly empowered women. Less empowered women had a significant odd of experiencing sexual violence [AOR:1.92(1.59–2.31)] than that highly empowered women. Relatively higher odds of physical violence were evident from southern [AOR: 2.10 (1.82–2.42)] and eastern [AOR: 1.75(1.51–2.02)] regions, however, sexual violence was highly associated with western [AOR: 1.21 (0.92–1.59)] part of India. Partner’s alcohol drinking was found to be an attributing factor for any form of violence, i.e., emotional violence [AOR: 2.34 (2.09–2.63)], physical violence[AOR: 2.76 (2.52–3.03)] sexual violence [AOR: 3.31 (2.83–3.88)] or severe violence [AOR: 3.38 (2.94–3.89)]. Partner controlling behaviour also evolved as a determining factor for any violence, i.e., emotional violence [AOR:6.63(5.87–7.47)], Physical violence [AOR:3.62(3.33–3.94)] and sexual violence [AOR:6.60(5.53–7.88)].

**Table 2:** Multivariate logistic regression analysis between socioeconomic characteristics with various forms of Intimate Partner violence

Background characteristics		Types of violence			
		Emotional AOR 95% CI	Physical AOR 95% CI	Sexual AOR 95% CI	Severe A.O.R. 95% CI
**Age Group**	15–24 years	Reference	Reference	Reference	Reference
	25–34 years	1.29** 1.10–1.51	1.47*** 1.30–1.66	1.31* 1.03–1.66	1.68*** 1.32–2.15
	≥ 35 years	1.43*** 1.21–1.69	1.55*** 1.38–1.75	1.49** 1.16–1.92	2.18*** 1.69–2.80
**Residence**	Urban	1.09 0.94–1.27	1.05 0.93–1.20	1.01 0.81–1.27	0.97 0.79–1.20
	Rural	Reference	Reference	Reference	Reference
**Caste**	Scheduled Caste	1.23** 1.07–1.40	1.11* 1.00-1.23	1.08 0.88–1.33	1.07 0.90–1.26
	Scheduled Tribe	1.03 0.86–1.24	0.97 0.85–1.11	0.85 0.67–1.08	0.73** 0.57–0.92
	Other Backward Class	Reference	Reference	Reference	Reference
	None of the casts	1.21* 1.03–1.41	0.83** 0.73–0.94	1.12 0.89–1.40	0.79 0.63-1.00
**Respondent Educational attainment**	No formal education	1.27 0.98–1.65	1.65*** 1.35–2.02	1.04 0.74–1.47	1.51* 1.07–2.13
	Completed primary education	1.13 0.86–1.48	1.50*** 1.22–1.85	1.04 0.73–1.48	1.57* 1.11–2.22
	Completed secondary education	1.27* 1.01–1.60	1.34** 1.11–1.62	0.99 0.73–1.36	1.42* 1.05–1.91
	Higher secondary and above	Reference	Reference	Reference	Reference
**Partner’s Educational attainment**	No formal education	1.33* 1.06–1.67	1.25* 1.04–1.51	1.30 0.93–1.81	1.37 0.97–1.93
	Completed primary education	1.35* 1.07–1.69	1.22* 1.01–1.46	1.35 0.99–1.83	1.26 0.89–1.79
	Completed secondary education	1.16 0.96–1.39	1.10 0.94–1.30	1.18 0.90–1.54	1.01 0.74–1.39
	Higher secondary and above	Reference	Reference	Reference	Reference
**Religion**	Hindu	1.19 0.86–1.65	1.87** 1.45–2.41	1.41 0.92–2.16	1.21 0.79–1.86
	Muslim	1.23 0.85–1.78	1.83** 1.37–2.44	2.25** 1.37–3.71	1.35 0.80–2.30
	Christian	Reference	Reference	Reference	Reference
	Other religions	1.05 0.62–1.78	1.50* 1.02–2.20	1.09 0.61–1.94	0.77 0.43–1.37
**Wealth Index**	Poorest quintile	1.36* 1.08–1.71	1.47*** 1.21–1.79	1.79** 1.23–2.58	1.60** 1.17–2.22
	Poorer quintile	1.25* 1.00-1.57	1.60*** 1.33–1.93	1.65** 1.17–2.33	1.57** 1.16–2.12
	Middle quintile	1.23* 1.00-1.50	1.38*** 1.16–1.64	1.23 0.89–1.70	1.40* 1.06–1.86
	Richer quintile	1.07 0.87–1.32	1.18* 1.00-1.39	1.16 0.84–1.60	1.22 0.92–1.60
	Richest quintile	Reference	Reference	Reference	Reference
**Region**	North	Reference	Reference	Reference	Reference
	Central	1.00 0.85–1.17	1.64*** 1.44–1.86	0.89 0.71–1.11	1.00 0.81–1.25
	East	1.34** 1.12–1.61	1.75*** 1.51–2.02	1.19 0.91–1.54	1.14 0.90–1.44
	North-east	1.15 0.91–1.46	1.60*** 1.35–1.89	1.14 0.86–1.50	0.93 0.69–1.25
	West	1.31** 1.08–1.60	1.34*** 1.14–1.57	1.21 0.92–1.59	1.22 0.93–1.59
	South	1.80*** 1.51–2.14	2.10*** 1.82–2.42	0.87 0.68–1.12	1.30* 1.03–1.63
**Women empowerment**	Less Empowered	1.52*** 1.32–1.75	1.20* 1.08–1.33	1.92*** 1.59–2.31	1.53*** 1.27–1.83
	Medium Empowered	1.17* 1.01–1.34	1.08 0.98–1.20	1.40** 1.14–1.73	1.07 0.88–1.29
	Highly Empowered	Reference	Reference	Reference	Reference
**Partner Drink Alcohol**	Yes	2.34*** 2.09–2.63	2.76*** 2.52–3.03	3.31** 2.83–3.88	3.38*** 2.94–3.89
	No	Reference	Reference	Reference	Reference
**Partner Controlling behaviours**	Yes	6.63*** 5.87–7.47	3.62*** 3.33–3.94	6.60** 5.53–7.88	6.08*** 5.16–7.15
	No	Reference	Reference	Reference	Reference

In the table or indication of p-value (i.e., *** *p* < 0.001, ** *p* < 0.01, * *p* < 0.05).

## Discussion

Our analysis showed a statistically significant increase in physical violence, particularly among women who were less empowered. At the individual level, it has been shown that women are less likely to experience IPV when they are more educated, higher income status, and are empowered. Household-level factors demonstrated that they had significance in intimate partner violence as well as the community-level factors showed the same (i.e., husband’s education, controlling behaviour and drinking Alcohol).

The results of this study demonstrate that a few individual factors strongly explain IPV. For instance, young women who belong to a scheduled caste, being from lower income group and with less level educationwere more likely to experience spousal violence. Previous evidence supported that higher prevalence of IPV is observed among women from Schdule Tribe and Schdeduled Caste [27, 28]. Being from lower socioeconomic status also found to be elevating the risk of IPV in women. The literature with the similar evidence confirm that the women from marginal poor segment of society [29–31] .

Significantly, the more alcohol is consumed, the more nuanced the association between the variables of women empowerment become. According to the findings of this study, women who indicate that their husbands frequently or occasionally consume alcohol have a higher likelihood of experiencing all types of IPV than empowered women who report their husbands never consume alcohol [33, 34].

Working women with higher education, on the other hand, experienced higher IPV exposures as compared to their non-working counterparts. The ego considerations of the spouses and gender prejudices in Indian society are likely reasons for any kind of violence [35–37]. This public health challenge can be addressed by enhancing economic empowerment there by could providing women the awareness and a platform for protest. Given that different levels of social ecology influence spousal violence, interventions at a higher level may be more effective in challenging spousal violence social norms rather than focusing on individual factors, which are difficult to change at the population level and may take decades or generations to be effective.

### Strength & limitation

This study used nationally representative data to understand the prevalence of intimate partner violence. It creates an aggregated index of women’s empowerment, providing a more comprehensive view of its relationship with IPV. The NFHS collects a large data set from a representative sample of the country and hence gives a good estimate of marital violence and its relationships with explanatory factors at the population level. However, one of the key drawbacks was its dependence on women’s self-reporting of partner violence. Spousal violence is delicate and intimate in nature, and it is difficult for women to divulge during major survey data collecting due to recall bias and fear of stigmatisation. Further, we were unable to validate the direction of causation and the causative mechanism of domestic and Intimate Partner violence in India using this cross-sectional data. In addition, our composite measure of women’s empowerment index was not strong by conventional statistical standards.

Finally, the implications of the findings are constrained because the data supplied only allowed for the examination of heterosexual relationships [39]. It should be emphasized, however, that monogamous heterosexual partnerships are the norm in India, signifying a larger reach in terms of generalizability.

### Implication

This study has numerous significant policy consequences. This study provides recent evidence for understanding the underlying factors of IPV in India, where wife-beating is high, women’s decision-making power is limited, and male-dominated cultures prevail across the country, though to varying degrees from rigid gender norms. Women’s empowerment, which in turn could ease the risk of IPV and domestic violence, may be enhanced by economic interventions such as conditional cash transfers gender sensitization workshops, media, and cultural campaigns and microcredit programs [40].

## Conclusion

The findings of this study highlight the need to enhance girls’ education, increasing women empowerment, equity in society by eliminating harmful socio-cultural practises. Nevertheless, sole reliance on economic empowerment falls short in ensuring the comprehensive protection of women. Interventions aimed at empowering women must engage with couples as units and operate at the community level, addressing issues of equal job opportunities and gender-specific roles to be effective.

### Electronic supplementary material

Below is the link to the electronic supplementary material.

Supplementary Material 1

## Data Availability

The dataset generated during and/or analyzed during the current study is available from the Demographic and Health Surveys (DHS) repository (with proper permission), Available at: https://www.dhsprogram.com/data/dataset/India_Standard-DHS_2020.cfm?flag=0.

## Abbreviations

NFHS: National family health survey
MoHFW: Ministry of health and family welfare
UT: Union territory
IPV: Intimate partner violence
SDG: Sustainable development goals
PCA: Principal component analysis
AOR: Adjusted odds ratio
CI: Confidence interval
OR: Odds ratio
WHO: World health organization
P.T.S.D.: Post-traumatic stress disorder
DHS: Demographic health survey
CAPI: Computer-assisted personal interview
