# Supplementary material for: Prevalence of intimate partner violence among Indian women and their determinants: a cross-sectional study from national family health survey – 5
Source: BMC Womens Health. 2024 Jun 22;24:363. doi: 10.1186/s12905-024-03204-x (PMC11193235; doi:10.1186/s12905-024-03204-x)
Supplement: Supplementary file 1 — Supplementary Material 1 [file 12905_2024_3204_MOESM1_ESM.docx]

**Table A1: Lists of variables selected for making women empowerment**

| Variable | Observation | Mean | Std. dev. | Min | Max |
| --- | --- | --- | --- | --- | --- |
| Decision Indicator | | | | | |
| Own healthcare | 60,480 | 0.82 | 0.38 | 0 | 1 |
| Large house purchase | 60,480 | 0.80 | 0.40 | 0 | 1 |
| Visit family & relatives | 60,480 | 0.82 | 0.38 | 0 | 1 |
| Spend husband earnings | 59,623 | 0.78 | 0.41 | 0 | 1 |
| without telling go outside | 68,949 | 0.82 | 0.39 | 0 | 1 |
| Beating Indicator | | | | | |
| Beat the child | 68,949 | 0.75 | 0.44 | 0 | 1 |
| Beat wife when argues | 68,949 | 0.79 | 0.41 | 0 | 1 |
| Beat when refusing sex | 68,949 | 0.87 | 0.31 | 0 | 1 |
| Beat if burn food | 68,949 | 0.87 | 0.34 | 0 | 1 |
| Controlling Indicator | | | | | |
| Allowed market | 68,949 | 0.59 | 0.49 | 0 | 1 |
| Allowed her | 68,949 | 0.53 | 0.49 | 0 | 1 |
| Allowed out | 68,949 | 0.52 | 0.50 | 0 | 1 |
| Five Economic Indicator | | | | | |
| Owner of house | 68,949 | 0.31 | 0.46 | 0 | 1 |
| Owner of land | 68,949 | 0.24 | 0.43 | 0 | 1 |
| Respondent currently  working | 68,949 | 0.28 | 0.45 | 0 | 1 |
| Account in bank or other institution | 68,949 | 0.80 | 0.39 | 0 | 1 |
| Owns a mobile phone | 68,949 | 0.58 | 0.49 | 0 | 1 |

**Table A2 : Kaiser-Meyer-Olkin measure of sampling adequacy**

| Variable | KMO |
| --- | --- |
| Component 1 | 0.5859 |
| Component 2 | 0.5808 |
| Component 3 | 0.5790 |
| Component 4 | 0.5699 |
| Component 5 | 0.6259 |
| Overall | 0.5837 |

**Table A3: Unweighted description of the study samples.**

| **Background characteristics** | | **Frequency (n, %)** |
| --- | --- | --- |
| **N=68,949** | |  |
| **Age Group**  (n=68,949) | **15-24 years** | 14566,21.13 |
|  | **25-34 years** | 26431,38.33 |
|  | **≥35 years** | 27952, 40.54 |
| **Residence**  (n=68,949) | **Urban** | 17191,24.93 |
|  | **Rural** | 51758,75.07 |
| **Caste**  (n=65,207) | **Scheduled Caste** | 12912,19.69 |
|  | **Scheduled Tribe** | 13415,20.57 |
|  | **Other Backward Class** | 26288,40.31 |
|  | **None of the casts** | 12592,19.31 |
|  | **Not sure of their caste** | 362,0.55 |
| **Respondent Educational Attainment**  (n=68,949) | **No formal education** | 17798,25.81 |
|  | **Completed primary education** | 9070,13.15 |
|  | **Completed secondary education** | 32102,46.56 |
|  | **Higher secondary and above** | 9979,14.47 |
| **Partner’s Educational Attainment**  (n=60,535) | **No formal education** | 10353,17.10 |
|  | **Completed primary education** | 8805,14.55 |
|  | **Completed secondary education** | 32598,53.85 |
|  | **Higher secondary and above** | 8585,14.18 |
| **Religion**  (n=68,949) | **Hindu** | 51628,74.88 |
|  | **Muslim** | 8527,12.37 |
|  | **Christian** | 5224,7.58 |
|  | **Other religions** | 3570,5.18 |
| **Wealth Index**  (n=68,949) | **Poorest quintile** | 14719,21.35 |
|  | **Poorer quintile** | 15387,22.32 |
|  | **Middle quintile** | 14310,20.75 |
|  | **Richer quintile** | 13077,18.97 |
|  | **Richest quintile** | 11456,16.62 |
| **Region**  (n=67,582) | **North** | 13401,19.83 |
|  | **Central** | 14734,21.80 |
|  | **East** | 11,264,16.67 |
|  | **North-east** | 10600,15.68 |
|  | **West** | 6833,10.11 |
|  | **South** | 10750,15.91 |
| **Women empowerment**  (n=59,623) | **Less Empowered** | 20081,33.68 |
|  | **Medium Empowered** | 19710,33.06 |
|  | **High Empowered** | 19832,33.26 |
| **Partner Drink Alcohol**  (n=60,480) | **Yes** | 16360,27.05 |
|  | **No** | 44120,72.95 |
| **Partner Controlling Behaviours**  (n=68,949) | **Yes** | 24727,35.86 |
|  | **No** | 44222, 64.14 |

**Table A4: Percent Distribution of Sampled Women According to Background Characteristics.**

| **Socioeconomic Characteristic** | | **No. of Women (%)** | **Chi-square p Value** |
| --- | --- | --- | --- |
| **Age group** | **15-24 years** | 2,161(14.84) | 0.000 |
|  | **25-34 years** | 7345(27.79) |  |
|  | **≥35 years** | 8568(30.65) |  |
| **Residence** | **Urban** | 3764(21.90) | 0.000 |
|  | **Rural** | 14310(27.65) |  |
| **Caste** | **Scheduled Caste** | 3987(30.88) | 0.000 |
|  | **Scheduled Tribe** | 3292(24.54) |  |
|  | **Other Backward Class** | 7486(28.48) |  |
|  | **None of the casts** | 2441(19.39) |  |
| **Respondent Educational attainment** | **No formal education** | 6606(37.12) | 0.000 |
|  | **Completed primary education** | 2918(32.17) |  |
|  | **Completed secondary education** | 7454(23.22) |  |
|  | **Higher secondary and above** | 1096(10.98) |  |
| **Partner’s Educational attainment** | **No formal education** | 4071(39.32) | 0.000 |
|  | **Completed primary education** | 3169(35.99) |  |
|  | **Completed secondary education** | 9168(28.12) |  |
|  | **Higher secondary and above** | 1614(18.80) |  |
| **Religion** | **Hindu** | 14443(27.98) | 0.000 |
|  | **Muslim** | 2122(24.89) |  |
|  | **Christian** | 882(16.88) |  |
|  | **Other religions** | 627(17.56) |  |
| **Wealth Index** | **Poorest quintile** | 5045(34.28) | 0.000 |
|  | **Poorer quintile** | 4586(29.80) |  |
|  | **Middle quintile** | 3779(26.41) |  |
|  | **Richer quintile** | 2913(22.28) |  |
|  | **Richest quintile** | 1751(15.28) |  |
| **Region** | **North** | 2235(16.68) | 0.000 |
|  | **Central** | 4367(29.64) |  |
|  | **East** | 3677(32.64) |  |
|  | **North-east** | 2306(21.75) |  |
|  | **West** | 1526(22.33) |  |
|  | **South** | 3621(33.68) |  |
| **Women empowerment** | **Highly Empowered** | 7137(35.54) | 0.000 |
|  | **Medium Empowered** | 5911(29.99) |  |
|  | **Less Empowered** | 4674(23.57) |  |
| **Partner Drink Alcohol** | **Yes** | 7527(46.01) | 0.000 |
|  | **No** | 10547(23.91) |  |
| **Partner Controlling behaviours** | **Yes** | 11726(47.42) | 0.000 |
|  | **No** | 6348(14.35) |  |

**Table A5 : Results of univariate logistic regression analysis between socioeconomic characteristics with various forms of domestic violence.**

| **Background characteristics** |  | **Types of violence** | | | |
| --- | --- | --- | --- | --- | --- |
|  |  | **Emotional**  **OR(p values)** | **Physical**  **OR(p values)** | **Sexual**  **OR(p values)** | **Severe**  **OR(p values)** |
| **Age Group** | 15-24 years | Reference | Reference | Reference | Reference |
|  | 25-34 years | 2.14(0.000) | 2.44(0.00) | 1.95(0.00) | 2.46(0.00) |
|  | ≥35 years | 2.42(0.000) | 2.84(0.00) | 2.13(0.00) | 3.25(0.00) |
| **Residence** | Urban | Reference | Reference | Reference | Reference |
|  | Rural | 1.43(0.00) | 1.56(0.00) | 1.67(0.00) | 1.67(0.00) |
| **Caste** | Scheduled Caste | 1.54(0.000) | 1.95(0.001) | 1.45(0.001) | 2.13(0.000) |
|  | Scheduled Tribe | 1.30 (0.003) | 1.69(0.000) | 1.24(0.060) | 1.62(0.000) |
|  | Other Backward Class | 1.70(0.047) | 1.62(0.000) | 1.97(0.064) | 1.74(0.000) |
|  | None of the casts | 1.42(0.02) | Reference | Reference | Reference |
|  | Not sure of their caste | Reference | 1.23(0.34) | 1.40(0.45) | 1.509(0.24) |
| **Respondent Educational attainment** | No formal education | 4.74(0.000) | 6.12(0.000) | 5.58(0.000) | 7.67(0.000) |
|  | Completed primary education | 3.74(0.000) | 4.66(0.000) | 4.92(0.000) | 6.22(0.000) |
|  | Completed secondary education | 2.78(0.000) | 2.85(0.000) | 2.85(0.000) | 3.44(0.000) |
|  | Higher secondary and above | Reference | Reference | Reference | Reference |
| **Partner’s Educational attainment** | No formal education | 2.73(0.00) | 3.17(0.000) | 3.48(0.000) | 3.94(0.000) |
|  | Completed primary education | 2.31(0.00) | 2.60(0.000) | 3.12(0.000) | 2.96(0.000) |
|  | Completed secondary education | 1.69(0.000) | 1.76(0.000) | 1.99(0.000) | 2.81(0.007) |
|  | Higher secondary and above | Reference | Reference | Reference | Reference |
| **Religion** | Hindu | 1.63(0.001) | 1.89(0.000) | 1.82(0.001) | 2.03(0.000) |
|  | Muslim | 1.53(0.009) | 1.59(0.000) | 2.20(0.000) | 1.78(0.004) |
|  | Christian | 1.16(0.447) | Reference | Reference | 1.24(0.398) |
|  | Other religions | Reference | 1.034(0.834) | 1.24(0.379) | Reference |
| **Wealth Index** | Poorest quintile | 2.72(0.000) | 3.30(0.000) | 4.28(0.000) | 4.09(0.000) |
|  | Poorer quintile | 2.22(0.00) | 2.84(0.000) | 3.17(0.000) | 3.12(0.000) |
|  | Middle quintile | 1.95(0.000) | 2.16(0.000) | 2.03(0.000) | 2.59(0.000) |
|  | Richer quintile | 1.47(0.000) | 1.58(0.000) | 1.58(0.001) | 1.82(0.000) |
|  | Richest quintile | Reference | Reference | Reference | Reference |
| **Region** | North | Reference | Reference | Reference | Reference |
|  | Central | 1.47(0.00) | 2.17(0.000) | 1.42(0.000) | 1.70(0.000) |
|  | East | 2.13(0.000) | 2.50(0.000) | 2.25(0.000) | 2.08(0.000) |
|  | North-east | 1.36(0.000) | 1.95(0.000) | 1.58(0.000) | 1.55(0.000) |
|  | West | 1.28(0.002) | 1.27(0.000) | 1.11(0.330) | 1.15(0.241) |
|  | South | 2.13(0.000) | 2.41(0.000) | 1.25(0.015) | 1.97(0.000) |
| **Women empowerment** | Less  Empowered | 1.89(0.000) | 1.81(0.000) | 2.52(0.000) | 2.37(0.000) |
|  | Medium Empowered | 1.38(0.000) | 1.29(0.000) | 1.41(0.001) | 1.31(0.000) |
|  | Highly Empowered | Reference | Reference | Reference | Reference |
| **Partner Drink Alcohol** | Yes | 2.88(0.000) | 3.29(0.000) | 3.46(0.000 | 4.09(0.000) |
|  | No | Reference | Reference | Reference | Reference |
| **Partner Controlling behaviours** | Yes | 9.31(0.000) | 5.39(0.000) | 9.97(0.000) | 9.17(0.000) |
|  | No | Reference | Reference | Reference | Reference |
